# SARS-CoV-2 vaccination may mitigate dysregulation of IL-1/IL-18 and gastrointestinal symptoms of the post-COVID-19 condition

**DOI:** 10.1038/s41541-024-00815-1

**Published:** 2024-02-05

**Authors:** Claudia Fischer, Edith Willscher, Lisa Paschold, Cornelia Gottschick, Bianca Klee, Sophie Diexer, Lidia Bosurgi, Jochen Dutzmann, Daniel Sedding, Thomas Frese, Matthias Girndt, Jessica I. Hoell, Michael Gekle, Marylyn M. Addo, Julian Schulze zur Wiesch, Rafael Mikolajczyk, Mascha Binder, Christoph Schultheiß

**Affiliations:** 1 Division of Medical Oncology, University Hospital Basel, Basel, Switzerland; 2 Laboratory of Translational Immuno-Oncology, Department of Biomedicine, University, and University Hospital Basel, Basel, Switzerland; 3 Department of Internal Medicine IV, Oncology/Hematology, Martin Luther University Halle-Wittenberg, Halle, (Saale) Germany; 4 Institute for Medical Epidemiology, Biometrics, and Informatics (IMEBI), Interdisciplinary Center for Health Sciences, Medical School of the Martin Luther University Halle-Wittenberg, Halle, (Saale) Germany; 5 I. Department of Medicine, University Medical Center Hamburg-Eppendorf, Hamburg, Germany; 6 Protozoa Immunology, Bernhard Nocht Institute for Tropical Medicine, Hamburg, Germany; 7 Mid-German Heart Center, Department of Cardiology and Intensive Care Medicine, University Hospital, Martin Luther University Halle-Wittenberg, Halle, (Saale) Germany; 8 Institute of General Practice and Family Medicine, Martin-Luther-University Halle-Wittenberg, Halle, (Saale) Germany; 9 Department of Internal Medicine II, Martin Luther University Halle-Wittenberg, Halle, (Saale) Germany; 10 Pediatric Hematology and Oncology, Martin Luther University Halle-Wittenberg, Halle, (Saale) Germany; 11 Julius Bernstein-Institute of Physiology, Faculty of Medicine, Martin Luther University Halle-Wittenberg, Halle, (Saale) Germany; 12 I. Department of Internal Medicine, University Medical Center Hamburg-Eppendorf, Hamburg, Germany; 13 German Center for Infection Research (DZIF), Partner Site Hamburg-Lübeck-Borstel-Riems, Braunschweig, Germany; 14 University Medical Center Hamburg-Eppendorf, Institute for Infection Research and Vaccine Development (IIRVD), Hamburg, Germany

**Keywords:** Viral infection, Viral infection, Vaccines

## Abstract

The rapid development of safe and effective vaccines helped to prevent severe disease courses after SARS-CoV-2 infection and to mitigate the progression of the COVID-19 pandemic. While there is evidence that vaccination may reduce the risk of developing post-COVID-19 conditions (PCC), this effect may depend on the viral variant. Therapeutic effects of post-infection vaccination have been discussed but the data for individuals with PCC remains inconclusive. In addition, extremely rare side effects after SARS-CoV-2 vaccination may resemble the heterogeneous PCC phenotype. Here, we analyze the plasma levels of 25 cytokines and SARS-CoV-2 directed antibodies in 540 individuals with or without PCC relative to one or two mRNA-based COVID-19 vaccinations as well as in 20 uninfected individuals one month after their initial mRNA-based COVID-19 vaccination. While none of the SARS-CoV-2 naïve individuals reported any persisting sequelae or exhibited PCC-like dysregulation of plasma cytokines, we detected lower levels of IL-1β and IL-18 in patients with ongoing PCC who received one or two vaccinations at a median of six months after infection as compared to unvaccinated PCC patients. This reduction correlated with less frequent reporting of persisting gastrointestinal symptoms. These data suggest that post-infection vaccination in patients with PCC might be beneficial in a subgroup of individuals displaying gastrointestinal symptoms.

## Introduction

Infection with the zoonotic severe acute respiratory syndrome coronavirus 2 (SARS-CoV-2) causes the coronavirus disease 2019 (COVID-19)^1,2^. Due to the multi-organ tropism of SARS-CoV-2, COVID-19 manifestations are often systemic and characterized by a broad severity spectrum with high morbidity and an elevated risk of mortality in distinct patient groups^1,3^. After the global spread of SARS-CoV-2 in 2020, the rapid development of several novel vaccine platforms within one year was key to mitigate the pandemic^4–8^. This unprecedented achievement was possible due to prior knowledge from the development and preclinical studies of vaccine candidates against SARS-CoV and Middle Eastern respiratory syndrome coronavirus (MERS-CoV) that identified the spike protein of human coronaviruses as the cardinal antigenic target to generate broad neutralizing B and T cell responses^4,9^. In August 2023, the two licensed mRNA-based COVID-19 vaccines BNT162b2 (BioNTech/Pfizer)^10^ and mRNA-1273 (Moderna)^11^, both of which encode a modified full-length SARS-CoV-2 S1 spike protein designed to stabilize the prefusion conformation, account for 90% of administered doses in the European Union and the United States^12^. Large clinical trials and real-world data clearly show that both vaccines are extremely safe and provide high protection against symptomatic and severe infection by eliciting neutralizing B and T cell responses including immunological memory that are also effective against different emerging variants of concern in single-dose or (heterologous) booster settings^13–28^. Notably, COVID-19 vaccination has similar risk and safety profiles in immunocompromised individuals^29^, patients with cancer^30–33^ or during pregnancy^34^.

Although vaccines are highly successful in reducing morbidity and mortality of acute COVID-19, their efficacy in preventing long-term consequences of a SARS-CoV-2 infection is less clear. Around 10-15% of COVID-19 patients have persisting health impairments beyond four weeks of symptom onset that are heterologous in their expression and can last for months with significant impairments for the quality of life^35–39^. For earlier variants of SARS-CoV-2 studies suggested that the likelihood of developing symptoms after infection (post-COVID-19 condition; PCC) is less frequent in individuals with pre-infection vaccination compared to those without^40–45^. Across studies, this effect was especially observed after two vaccine doses in early phases of the pandemic^46^. In contrast, studies on vaccination effects after PCC diagnosis are rare, of smaller cohort size, and do not report clear benefits^47–50^. These studies mostly lack quantifiable biomarkers for disease activity and analyze PCC individuals as a homogenous group. More and more data, however, suggest that PCC encompasses several pathophysiological subclasses that might respond differently to post-infection vaccination^51–59^. In addition, manifestations of rare vaccination side effects resemble the heterogenous, and in individual cases also persisting, symptoms of PCC. While this post-vaccination phenotype is now recognized as a clinical entity and research on its molecular underpinnings is ongoing, its similarity to PCC has also been exploited to spread misinformation on vaccine safety and drive vaccine hesitancy^60,61^.

We compared two COVID-19 vaccination groups to detect immune changes related to PCC. One group included 20 SARS-CoV-2 naïve healthcare workers, and the other 540 people with varying infection history and known PCC status. Single or booster doses resulted in strong antibody responses without causing cytokine dysregulation or worsening symptoms. Notably, those with ongoing PCC symptoms that were vaccinated showed lower IL-1β and IL-18 levels and lesser gastrointestinal symptoms. This data confirms vaccination safety and efficacy while pointing at PCC patients that may benefit from post-infection vaccination.

## Results

### Characterization of the SARS-CoV-2 naïve vaccination cohort

Between December 2020 and January 2021 at the beginning of the German COVID-19 vaccination campaign, we recruited 20 healthcare workers with a median age of 39.5 (range 29-58) at the University Hospital Halle (Saale) Germany who were never tested positive for SARS-CoV-2, to monitor vaccine efficiency during the initial rollout. We used this cohort to probe for potential plasma cytokine dysregulations four weeks after their initial mRNA-based vaccine dose. Of these 20 individuals, 19 received the BioNTech-Pfizer vaccine BNT162b2 and one received the Moderna mRNA-1273 vaccine. To benchmark vaccination-induced S1-IgG titers, we used 20 hospitalized individuals with acute COVID-19 and 20 individuals who recently recovered after moderate disease. These individuals were sampled between April and June 2020 as part of the HACO cohort at the University Hospital Halle (Saale)^62,63^. The acute COVID-19 cohort included 10 individuals who required ICU from which 7 individuals finally succumbed to the disease. As a vaccination control cohort, we included 11 healthcare workers without a history of SARS-CoV-2 infection and a median age of 37 (range 26-58) who received the seasonal influenza vaccine (VaxigripTetra 2020/2021) between October and November 2020 at the same hospital. The demographic characteristics of all cohorts are listed in Table 1.

**Table 1:** Characteristics of COVID-19 and influenza vaccination cohorts.

	COVID-19 vaccination	Influenza vaccination	acute COVID-19	recovered COVID-19
Nb of participants	20	11	20	20
Sex				
Female	12 (60%)	7 (63.6%)	12 (60%)	11 (55%)
Male	8 (40%)	4 (36.4%)	8 (40%)	9 (45%)
Age (years)				
Median age all	39.5	37	67.5	37
Range	29-58	26-58	23-80	23-68
Median age females	41	38	69	33
Range	31-58	29-58	23-80	23-62
Median age males	33	33.5	66	37
Range	29-48	26-49	29-80	33-68
Vaccine				
BNT162b2 (BioNTech-Pfizer)	19 (95%)			
mRNA-1273 (Moderna)	1 (5%)			
VaxxigripTetra2020/2021 (Sanofi)		11 (100%)		
Comorbidities				
Arterial hypertension	4 (20%)		11 (55%)	1 (5%)
Asthma	3 (15%)			1 (5%)
Hematol. malignancy			3 (15%)	
Diabetes			4 (20%)	1 (5%)
COPD			2 (10%)	
Neurodermatitis	1 (5%)			
Hypothyroidism	1 (5%)		4 (20%)	
Sampling after vaccination/first symptoms (days)				
Median	30	28	15.5	42
Range	23-32	14-28	3-40	23-80

### mRNA-based COVID-19 vaccination elicits robust humoral SARS-CoV-2-directed immune responses but no persisting cytokine dysregulations

**Fig. 1:**
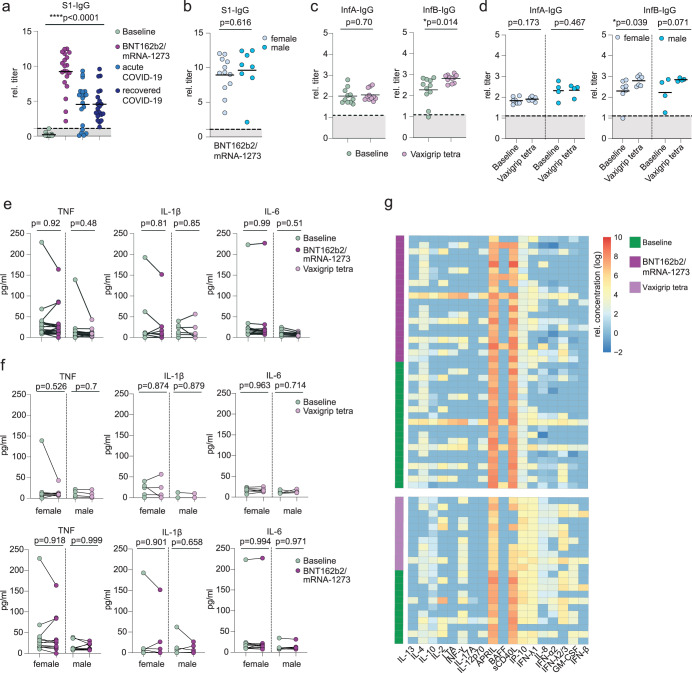
Administration of mRNA-based COVID-19 vaccines induces robust antibody responses but no cytokine dysregulation in SARS-CoV-2 naïve individuals. **a** Mean relative titers of SARS-CoV-2 S1-specific IgG antibodies in the plasma of healthy individuals without prior SARS-CoV-2 infection on the day of and four weeks after vaccination with one dose of BNT162b2 (*n* = 19) or mRNA-1273 (*n* = 1) vaccine. Patients with acute COVID-19 (*n* = 20) or after recovery (*n* = 20) were used as controls. Cohort characteristics in Table 1. Dotted lines indicate signal threshold for positivity. **b** Vaccination-induced antibody titers from (a) separated in females (*n* = 12) and males (*n* = 8). **c** Mean relative titers of IgG class antibodies directed against influenza A and B strains in healthy individuals (*n* = 11) on the day of and four weeks after vaccination with the seasonal influenza vaccine VaxigripTetra 2020/2021. **d** Vaccination-induced anti-influenza A/B antibody titers from (**c**) separated in females (*n* = 7) and males (*n* = 4). **e** Plasma levels of TNF, IL-1β and IL-6 after COVID-19 or influenza vaccination. **f** Sex-dependent plasma levels of TNF, IL-1β and IL-6 after COVID-19 or influenza vaccination. **g** Heatmap of plasma levels of indicated soluble factors after COVID-19 or influenza vaccination. Concentrations were plotted after log-transformation. Statistics in panels a-f: two-sided t-test.

To assess the humoral immune response in SARS-CoV-2 naïve individuals after their initial mRNA-based COVID-19 vaccination, we first quantified circulating IgG class antibodies directed against the SARS-CoV-2 S1 protein. While none of the included probands had detectable virus-specific antibody titers on the day of vaccine administration, all tested individuals had robust S1-IgG levels at a median of 30 days (range 23-32) post-COVID-19 vaccination (Fig. 1a). Notably, vaccine-induced S1-IgG titers exceeded titers observed in patients with acute COVID-19 or after early recovery (Fig. 1a; Table 1). We did not observe sex-dependent differences in S1-IgG titers (Fig. 1b). None of the participating individuals reported persisting sequelae four weeks post-vaccination. Although less prominent, we observed a similar sex-independent pattern for influenza B-directed IgG antibodies in the control cohort at a median of 28 days (range 14-28) post-influenza vaccination (Fig. 1c, d). Next, we quantified plasma levels of TNF, IL-1β, and IL-6 which are consistently described across studies as dysregulated in individuals after SARS-CoV-2 infection, especially in those with ongoing PCC symptoms^47,53,64^. As shown in Fig. 1e, f, we did not detect any general or sex-dependent systemic enrichment of these factors four weeks after vaccination. The same pattern was found in the control cohort after vaccination with the seasonal inactivated influenza vaccine (Fig. 1e, f). We also quantified the levels of 18 additional plasma factors that have been associated with PCC in distinct patient subsets^55^. None of them showed any pattern of systemic dysregulation four weeks after COVID-19 or influenza vaccination (Fig. 1g).

### Vaccination status and SARS-CoV-2 antibody titers of the post-acute COVID-19 cohort

**Fig. 2:**
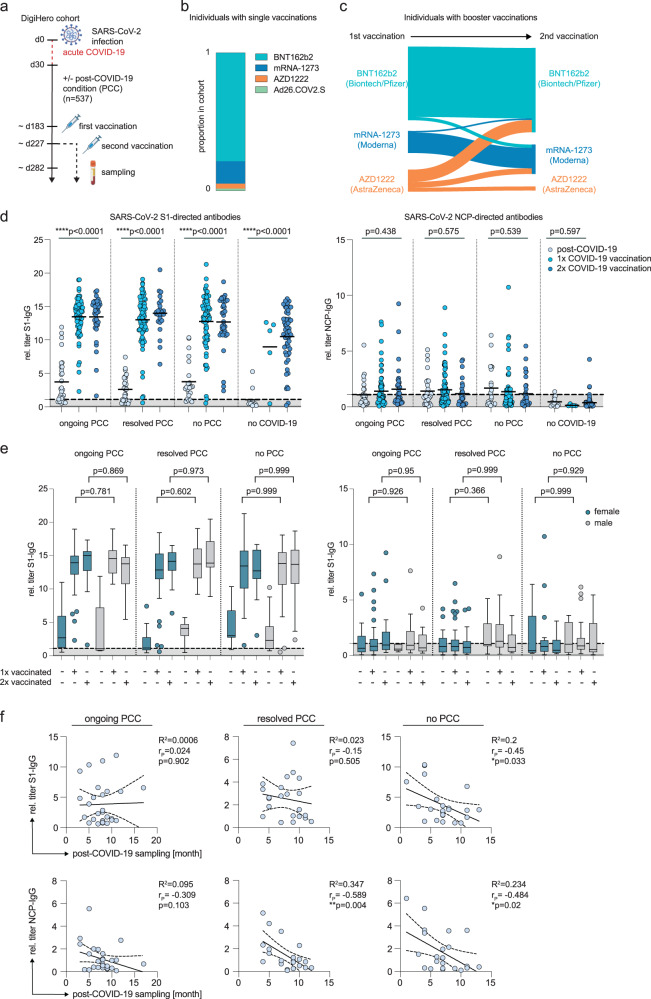
Vaccination after SARS-CoV-2 infection boosts spike-specific antibody titers independent of persisting sequelae. **a** Schematic representation of vaccination and sampling of the DigiHero cohort. **b** Proportion of administered vaccines in individuals with single-dose vaccinations. **c** Migration plot indicating distribution of administered vaccines in individuals who received two post-infection vaccine doses before sampling. **d**, **e** Relative S1 and NCP-specific IgG antibody titers in post-infection PCC-related groups and uninfected individuals plotted as means with respect to vaccination status (**d**) or as Tukey’s box-plot with respect to vaccination status and sex (**e**). In **e**, the centre line represents the median, lower bounds of boxes indicate the first quartile, upper bound the third quartile. Whiskers indicate the 1.5 interquartile range (IQR), dots above or below whiskers indicate data points outside 1.5 IQR. Dotted lines indicate signal threshold for positivity. Group sizes: Individuals with ongoing PCC who were unvaccinated (*n* = 30, 25 females, 5 males), received one vaccination (*n* = 72, 50 females, 22 males) or two vaccinations (*n* = 37, 24 females, 13 males); Individuals who resolved PCC and were unvaccinated (*n* = 29, 19 females, 10 males), received one vaccination (*n* = 76, 48 females, 28 males) or two vaccinations (*n* = 26, 15 females, 11 males); Individuals who never reported PCC and were unvaccinated (*n* = 23, 11 females, 12 males), received one vaccination (*n* = 69; 32 females, 37 males) or two vaccinations (*n* = 34; 17 females, 17 males); Individuals without COVID-19 who were unvaccinated (*n* = 12), received one vaccination (*n* = 5) or two vaccinations (*n* = 56). Statistics: Group comparisons were performed using ordinary one-way ANOVA followed by post-hoc testing (Tukey’s multiple comparisons test). The calculated p values for ANOVA are indicated in (**d**), p values in (**e**) represent post hoc analyses for female vs mal comparisons. **f** Linear regression and Pearson correlation analysis for S1-IgG or NCP-IgG titers and sampling time point in months after first symptoms of COVID-19 for unvaccinated individuals with ongoing PCC (*n* = 29), who resolved PCC (*n* = 22) or who never reported PCC (*n* = 23). Correlation coefficient R^2^, Pearson correlation coefficients (r_P_) and p values are indicated.

While we did not observe any systemic PCC-like cytokine perturbations after vaccination in SARS-CoV-2 naïve individuals, we next tested whether post-infection vaccination has an impact on the systemic cytokine landscape in individuals with or without ongoing PCC symptoms. To address this question, we selected 540 individuals from the ongoing DigiHero cohort study^47,55^ for which plasma cytokine or SARS-CoV-2 antibody levels were available and who provided sufficient answers on the online questionnaire to assess their vaccination and PCC status. All individuals of this cohort who reported SARS-CoV-2 infection contracted the virus prior to vaccine rollout. The median time for study participation relative to positive PCR or antigen test was 8 months (range 1-20). Of these 540 individuals, 439 ( = 82%) received at least one COVID-19 vaccine dose (Table 2). The initial vaccinations were performed at a median of 183 days (range 21-433) after the first positive COVID-19 test, the first booster vaccinations were performed at a median of 44 days (range 21-210) after the initial vaccination (Fig. 2a). Blood sampling was performed at a median of 100 days (range 10-330) after the last vaccination (Fig. 2a). At the time of sampling, the majority of individuals received at least one mRNA-based vaccination: From those individuals with single vaccination, 95% either received the BNT162b2 (79%) or mRNA-1273 (16%) vaccine (Fig. 2b), from those individuals with two vaccinations, 81% had received at least one mRNA-based vaccination as booster (Fig. 2c).

**Table 2:** Characteristics of PCC cohort.

	ongoing PCC	resolved PCC	no PCC	no COVID-19
Total n	162	146	136	96
Sex				
female	116 (71.6%)	91 (62.3%)	61 (44.9%)	53 (55.2%)
male	46 (28.4%)	55 (36.7%)	75 (55.1%)	43 (44.8%)
Age (years)				
median age all	50	47	45	44
range	20-85	18-81	18-78	18-87
median age females	49.5	45	46.5	46
range	20-84	18-81	20-75	18-72
median age males	50.5	52	44	40.5
range	19-85	18-76	18-78	18-87
Vaccination status				
unvaccinated	30 (18.5%)	32 (21.9%)	24 (17.6%)	16 (16.7%)
1x vaccinated	84 (51.9%)	85 (58.2%)	76 (55.9%)	6 (6.3%)
2x vaccinated	48 (29.6%)	29 (19.9%)	36 (26.5%)	74 (77%)
Sampling time point after first symptoms (months)				
median	8	8	8	
range	2-20	3-19	2-19	
Time between blood sampling and last vaccination (days)				
median all vaccinations	83	92	111	116
range	11-224	10-295	11-330	10-270
median one vaccination	95	124	119	95
range	11-209	10-270	16-212	14-245
median two vaccinations	72	126	107	116
range	12-224	11-295	11-330	10-270

For analysis, we defined individuals with ongoing PCC symptoms (*n* = 162), individuals who resolved PCC (*n* = 146) and individuals who never reported PCC (*n* = 136) as separate post-infection outcome groups (Table 2). Individuals who never had a positive COVID-19 test (*n* = 96) served as control (Table 2). Quantification of plasma antibodies directed against the S1 and NCP proteins of SARS-CoV-2 validated the provided information from the online questionnaire (Fig. 2d). Notably, 3 individuals who never tested positive for COVID-19 had detectable levels of S1 and/or NCP-directed antibodies arguing for the presence of cross-reactive antibodies originating from infections with other coronaviruses or asymptomatic infection with SARS-CoV-2 (Fig. 2d). The sequential administration of two vaccine doses resulted in a step-wise increase of the mean S1-IgG levels in individuals without prior SARS-CoV-2 infection. In line with (Fig. 1a), these levels were already higher after one vaccine dose as compared to the S1-IgG titers in recovered COVID-19 patients (Fig. 2d). In patients with prior SARS-CoV-2 infection, one round of vaccination substantially (3-4 fold) boosted S1-IgG titers independent of PCC status above the levels observed in unvaccinated post-COVID-19 individuals (Fig. 2d). We did not observe a further increment of S1-specific IgG antibodies mediated by a second vaccination (first booster vaccination) around one month after receiving the first dose, although individuals who resolved PCC symptoms appeared to have higher mean levels after the second dose (two-sided unpaired t-test: p = 0.22; Fig. 2d). Notably, the mean S1-IgG levels in vaccinated post-infection individuals were generally higher than those observed in uninfected individuals after two consecutive vaccinations (Fig. 2d). We did not observe any sex-specific patterns of SARS-CoV-2 antibody titers in any of the different PCC or non-PCC groups (Fig. 2e). Next, we performed correlation and linear regression analyses in unvaccinated individuals with prior SARS-CoV-2 infection to test for differences in the longitudinal antibody kinetics within the different PCC groups. We observed a clear decay of NCP-IgG antibodies over time in individuals who resolved PCC or never reported it (Fig. 2f). Individuals with ongoing PCC trended in the same direction but less clearly (Fig. 2f). Interestingly, waning S1-IgG titers were observed in individuals who never reported PCC or with resolved PCC (Fig. 2f), but individuals with ongoing PCC symptoms appeared to have more stable S1-IgG titers over time (Fig. 2f).

### Post-infection vaccination is associated with lower levels of IL-1β and IL-18 in patients with ongoing PCC symptoms

**Fig. 3:**
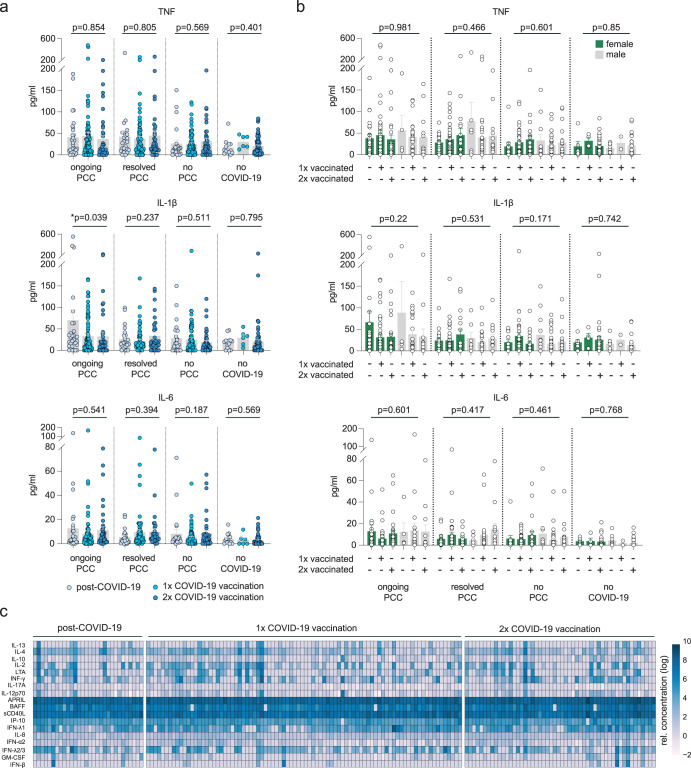
Quantification of post-infection plasma cytokine levels relative to vaccination status. **a**, **b** Mean plasma levels of TNF, IL-1β and IL-6 with respect to vaccination status (**a**) or vaccination status and sex (**b**). Error bars indicate ± SEM. Group sizes: Individuals with ongoing PCC who were unvaccinated (*n* = 29, 24 females, 5 males), received one vaccination (*n* = 83, 58 females, 25 males) or two vaccinations (*n* = 43, 30 females, 13 males); Individuals who resolved PCC and were unvaccinated (*n* = 24, 17 females, 7 males), received one vaccination (*n* = 68, 40 females, 28 males) or two vaccinations (*n* = 32, 18 females, 14 males); Individuals who never reported PCC and were unvaccinated (*n* = 24, 12 females, 12 males), received one vaccination (*n* = 78; 36 females, 42 males) or two vaccinations (*n* = 41; 21 females, 20 males); Individuals without COVID-19 who were unvaccinated (*n* = 14), received one vaccination (*n* = 6) or two vaccinations (*n* = 65). Statistics: Group comparisons were performed using ordinary one-way ANOVA followed by post-hoc testing (Tukey’s multiple comparisons test). **c** Heatmap of indicated plasma cytokine levels in patients with ongoing PCC with or without vaccination. Concentrations were log-normalized before plotting.

Since symptomatic PCC is associated with elevated levels of pro-inflammatory cytokines and chemokines^47,51,55,64–66^, we next asked whether post-infection vaccination affects the levels of these factors. For analysis, we choose a panel of 21 soluble factors^47^. First, we again focused on TNF, IL-1β, and IL-6 given their recurrent reporting in the context of PCC pathology. As shown in Fig. 3a, post-infection vaccination did not affect plasma levels of TNF and IL-6, irrespective of the number of doses or PCC status. However, plasma levels of IL-1β were lower in individuals with ongoing PCC who received one or two vaccine doses after infection (Fig. 3a). Notably, the detected plasma patterns were equally found in female and male participants (Fig. 3b). In addition, we also tested for vaccination-associated effects on the levels of IL-2, IL-4, IL-8, IL-10, IL-12p70, IL-13, IL-17A, IFN-α2, IFN-β, IFN-γ, IFN-λ1, IFN-λ2/3, IP-10, GM-CSF, LTA (TNF-β), soluble (s)CD40L, APRIL, and BAFF. None of these factors did exhibit any vaccination-associated concentration differences in the plasma of patients with ongoing PCC (Fig. 3c).

**Fig. 4:**
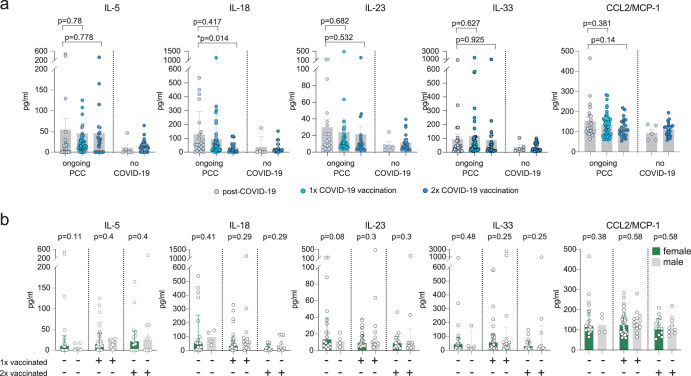
Profiling of monocyte/macrophage-related plasma factors in PCC relative to vaccination status. **a**, **b** Mean plasma levels of indicated cytokines. Error bars indicate ± SEM. Group sizes: Individuals with ongoing PCC who were unvaccinated (*n* = 22, 18 females, 4 males), received one vaccination (*n* = 45, 32 females, 13 males) or two vaccinations (*n* = 22, 13 females, 9 males); Individuals without COVID-19 who were unvaccinated (*n* = 5) or received two vaccinations (*n* = 23). Statistics: Two-sided t-test with Welch’s correction.

Next, we extended our analysis to a subset of patients with ongoing PCC symptoms for which levels of IL-5, IL-9, L-17F, IL-18, IL-22, IL-23, IL-33, CCL2/MCP-1, soluble CD206 (MMR), and soluble CD163 were available. These cytokines were initially selected to assess different activation states of monocytes and macrophages which most likely represent the main source of systemic TNF, IL-1β, and IL-6 elevations^47^. We restricted our analysis to IL-5, IL-18, IL-23, IL-33, and CCL2/MCP-1, because these factors displayed the clearest increase in plasma levels post-COVID-19^55^. While we did not detect vaccination-associated differences in plasma levels for IL-5, IL-23, IL-33, and CCL2/MCP-1, we observed a step-wise reduction of IL-18 levels after post-infection vaccinations in individuals with ongoing PCC (Fig. 4a). Notably, two post-infection vaccinations reduced the mean levels of IL-18 to the levels observed in individuals without COVID-19 (Fig. 4a). We did not notice any sex-dependent differences in cytokine levels after vaccination (Fig. 4b).

### Reduced levels of IL-1β and IL-18 in patients with ongoing PCC symptoms are associated with less frequent reporting of gastrointestinal sequelae

**Fig. 5:**
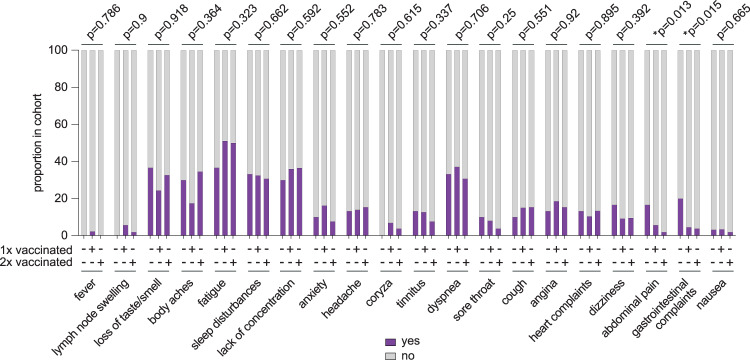
Frequency of self-reported symptoms in patients with ongoing PCC after vaccination. Indicated self-reported symptoms were retrieved from the DigiHero data set and analyzed with respect to vaccination status. Only symptoms that were reported as moderate or severe were considered to be symptomatic. Groups: Ongoing PCC, not vaccinated (*n* = 30); ongoing PCC, one time vaccinated post-infection (*n* = 86); ongoing PCC, two times vaccinated post-infection (*n* = 52). Statistics: Chi-squared test for trend in proportions.

Finally, we assessed whether the observed reduction of IL-1β and IL-18 after vaccination is associated with differential reporting of distinct PCC-associated symptoms by affected patients. The DigiHero cohort study includes an online questionnaire that asks for symptoms and their severity in patients with ongoing PCC^47^. We retrieved the information for the 20 most common symptoms from this data set and analyzed their occurrence dependent on vaccination status. For analysis with sufficient statistical power, we only considered patients who reported post-COVID-19 sequelae as moderate or severe to be symptomatic. Notably, we did not observe a correlation of cytokine levels with the number of reported PCC symptoms (Supplementary Fig. 1). As shown in Fig. 5, vaccination status had no impact on most symptoms in patients with ongoing PCC. Nevertheless, we noted less frequent reporting of abdominal pain and gastrointestinal complaints after post-infection vaccination (Fig. 5). This effect was equally observed after the administration of one and two vaccine doses (Fig. 5). Notably, the fraction of patients with ongoing PCC that also reported gastrointestinal symptoms (*n* = 13) displayed independent of vaccination slightly increased (one-sided t-test p = 0.187) mean plasma levels of IL-1β (55.6 pg/ml, range 0-354.5 pg/ml) as compared to those patients with ongoing PCC that did not report gastrointestinal symptoms (37.6 pg/ml, range 0-554.9 pg/ml). In case of IL-18, mean plasma levels for PCC patients with gastrointestinal symptoms were 70.9 pg/ml (range 0-452.6 pg/ml) for those without 89 pg/ml (range 0-1141.9 pg/ml) (p = 0.37).

## Discussion

The COVID-19 pandemic has spurred a worldwide effort to develop effective vaccines against the SARS-CoV-2 virus at unprecedented speed from which more than 20 are currently authorized for use^67^. In Europe and the US, the predominant vaccines in use are either based on adenoviral vectors (AZD1222 from AstraZeneca^5^ and Ad26.COV2.S from Johnson&Johnson^6^) or mRNA (BNT162b2 from BionNTech-Pfizer^7^ and mRNA-1273 from Moderna^8^). Numerous large clinical trials and real-world data with more than 9 billion doses administered in more than 180 countries until August of 2023 (WHO data) have demonstrated the effectiveness and safety of these vaccines. As vaccination campaigns progress, it has become imperative to understand the potential effects of vaccination on individuals who have previously experienced COVID-19 and those who may be susceptible to PCC. This is also true for the characterization of rare vaccine-induced adverse events.

The presented study contributes to specific aspects of vaccine effectiveness and safety. All included vaccinees mounted high titers of SARS-CoV-2 S1-specific IgG antibodies after one dose of mRNA-based vaccination, while none developed persisting adverse sequelae four weeks or later after vaccine administration. Consistent with published data^68,69^, we observed that one dose of mRNA-based COVID-19 vaccination generates higher titers of S1-IgG antibodies as compared to infection, showcasing the high efficiency of these vaccines to induce protective immunity. In addition, the described antibody profiles support prior knowledge that shows the cumulative benefit of booster vaccinations or hybrid immunity for generating high antibody quantities with immunogenetic breadth towards different SARS-CoV-2 variants of concern as well as functional B and T cell memory^16,70,71^. It should be noted that increased antibody quantity in hybrid immunity, as also observed here, is not necessarily linked to increased quality, although antibody titers can serve as a correlate for vaccine-induced protection^72–74^. Triple vaccination can outperform immunological settings of virus-induced immunity with respect to the breadth of antibody reactivity towards viral variants^70^. The mechanical basis of this observation might be the deficiency of germinal centers during acute infection which is not observed after vaccination^22,75,76^. Irrespective of the need to define adequate immunization schedules in this context and a rapidly evolving epidemiological landscape, the available vaccines provide highly effective protection from severe disease courses after infection with currently circulating variants of concern, including Omicron sublineages^15,16,19,77^.

Numerous studies have illuminated the complex relationship between SARS-CoV-2 infection and the potential for prolonged symptoms, commonly referred to as PCC^35–39^. PCC manifests as a multisystemic disease with a broad symptom and severity spectrum. This diversity most likely originates from different mutually not exclusive pathophysiological routes and thus defines different molecular disease subsets. For instance, viral RNA or protein is recurrently found in the respiratory tract, the gut, the brain, kidney and circulates in the blood months after acute disease and might fuel ongoing SARS-CoV-2-specific or superantigenic immune responses in PCC patients^1,65,78–86^. This is also in line with our data that shows stable S1-IgG titers in unvaccinated patients with ongoing PCC months after acute COVID-19, while the levels of SARS-CoV-2-directed antibodies wane in all other analyzed unvaccinated post-infection individuals. Together with the observation that pre-vaccinated individuals are less likely to report persisting sequelae, PCC may especially develop in settings of low-efficiency immune responses that fail to clear all viral components while the refined immune response after vaccination facilitates efficient elimination of SARS-CoV-2^40–44,46^.

Our data, however, does not provide clear evidence that patients with ongoing PCC who were predominantly infected with the ancestral or alpha variants of SARS-CoV-2 overall significantly benefit from vaccination regarding their sequelae. Nevertheless, we observed that PCC patients who were vaccinated post-infection less frequently reported gastrointestinal symptoms and had lower levels of IL-1β and IL-18. These findings were remarkable for two reasons: First, the gut emerges as a major reservoir for persisting viral antigen in PCC^78,85,87,88^ and this persistence is associated with low levels or delayed production of spike-directed antibodies during acute COVID-19^89,90^. In addition, a recent publication demonstrated the role of increased intestinal IL-1β as a mediator of protective SARS-CoV-2-directed immunity and its association with lower loads of viral RNA within the intestine^91^. Thus, it seems plausible that the high levels of IL-1β in PCC reflect this protective role and that post-infection vaccination provides a sufficient refinement of the SARS-CoV-2-directed immune response to clear persisting viral antigens, at least in a subset of PCC patients. Given its overlapping cellular functions with IL-1β, especially in the context of the inflammasome, IL-18 may play a similar context-dependent role^92^. Nevertheless, this requires further investigation given the lack of longitudinal symptom reporting or plasma sampling in our study.

Finally, it is important to state that the data presented in this manuscript does not provide any hints that post-infection vaccination might exacerbate PCC-like symptoms or adversely impact individuals with earlier infection. We also did not detect any PCC-like signature in uninfected individuals after initial mRNA-based vaccination, neither did we notice any general imprint that could be interpreted as priming for potential PCC development after booster vaccination. Our data suggests that vaccination in PCC patients may play a role in ameliorating post-infection gut-related symptoms via IL-1 family cytokines. While we did not observe any vaccination-associated adverse events, these can affect a very small number of vaccinees and require larger studies. It is nevertheless important to acknowledge virus-induced and vaccine-induced pathology as distinct entities to understand their underpinnings and develop targeted treatments.

Together, our study corroborates the safety and efficiency of mRNA-based COVID-19 vaccines in SARS-CoV-2 naïve individuals or after infection. In addition, we provide biomarker-based data that suggests a benefit of post-infection vaccination for patients with PCC who suffer from gastrointestinal sequelae. This finding invites further exploration into the intricate interplay between vaccination, cytokine modulation, and gastrointestinal health.

## Methods

### Recruitment and sampling of vaccination cohorts

Twenty SARS-CoV-2 naïve healthcare workers from the University Hospital Halle (Saale), Germany, were recruited between December 2020 and January 2021 at the beginning of the German COVID-19 vaccination campaign to study vaccination efficiency after the initial mRNA vaccine rollout. Of these 20 individuals, 19 received the BioNTech-Pfizer Vaccine BNT162b2 (Tozinameran/Comirnaty), one the Moderna mRNA-1273 (Elasomeran/Spikevax) vaccine. As vaccination control, we used a cohort of additional 11 healthcare workers who received the seasonal influenza vaccine (VaxigripTetra 2020/2021) between October and November 2020 at the University Hospital Halle (Saale). Blood sampling of these vaccination cohorts was performed on the day of vaccine administration and four weeks later. The demographic characteristics of both vaccination cohorts are listed in Table 1. To assess the effect of post-infection vaccination on symptoms of post-COVID-19 condition, we used 540 individuals from the cohort study for digital health research in Germany (DigiHero) including 96 individuals without prior SARS-CoV-2 infection. Individuals were recruited between August 2021 and February 2022 via mailed invitation^47^. Participants completed an online questionnaire focusing on the detection and course of acute COVID-19, its sequelae, and vaccination status. Blood from the DigiHero participants was sampled once at a median of 8 months after the onset of the first COVID-19 symptoms. Demographic characteristics are listed in Table 2. The study was approved by the institutional review board (approval numbers approval number 2020-039 and 2020-076) and conducted in accordance with the ethical principles stated by the Declaration of Helsinki. Informed written consent was obtained from all participants or legal representatives. Plasma was isolated from whole blood via centrifugation of whole blood for 15 minutes at 2000 × g, followed by centrifugation at 12,000 × g for 10 minutes. All plasma samples were stored at - 80 °C before further use.

### SARS-CoV-2 and influenza antibody profiling

Relative titers of antibodies targeting the S1 domain of the spike (S) protein and the nucleocapsid protein (NCP) of SARS-CoV-2 were determined using the Anti-SARS-CoV-2-ELISA IgA/IgG and Anti-SARS-CoV-2-NCP-ELISA kits from Euroimmun (Lübeck, Germany). ELISAs were coated with the respective recombinant antigen. To determine the relative titers of IgG class antibodies directed against influenza A and B, we used the Anti-Influenza-A-Virus-ELISA (IgG) and Anti-Influenza-B-Virus-ELISA (IgG) ELISA Kits from Euroimmun. Influenza A ELISA plates were coated with inactivated influenza A strains (Texas, H3N2; Singapore, H1N1; California, H1N1 (Porcine Influenza)) isolated from the allantoic fluid of infected chick embryos. In the case of influenza B, plates were coated with the inactivated virus of the B/Hong Kong/5/72 variant. Assays were performed according to the manufacturer’s instructions. Readouts were performed at 450 nm using a Tecan Spectrophotometer SpectraFluor Plus (Tecan Group Ltd., Männedorf, Switzerland).

### Quantification of soluble factors in human plasma

Plasma cytokines were quantified using the LEGENDplex Human B Cell Panel (13-plex) and the Human Anti-Virus Response Panel (13-plex) (BioLegend) according to the manufacturer’s instructions. In addition, plasma levels of IL-5, IL-18, IL-23, IL-33, and CCL2/MCP-1 were quantified using the respective capture beads and corresponding detection antibodies from the LEGENDplex Human Inflammation Panel (Cat. No. 740809) and Human Th Panel (Cat. No. 741027) (BioLegend). Read out of the LEGENDplex assays was performed on a BD FACSCelesta. Concentrations were calculated using the LEGENDplex cloud-based Qognit Data Analysis Software (BioLegend). Heatmap of log-transformed plasma levels were generated with the R package pheatmap using the R version 4.3.1 and RStudio 2023.06.1.

### Statistical analysis

Differences in plasma levels of antibodies or cytokines between the two groups were studied using the unpaired two-sided t-test. Comparisons between multiple groups were performed using ordinary one-way ANOVA followed by post-hoc testing (Tukey’s multiple comparisons test). The association between categorial symptom reporting and the number of post-infection vaccinations was tested with the chi-squared test for trend in proportions. All statistical analyses as well as the linear regression and Pearson correlation analyses for antibody plasma levels over time were performed using GraphPad PRISM 9.5.1 (GraphPad Software, La Jolla, CA, USA). Heatmaps were generated with the package pheatmap using R version 4.3.1 and RStudio 2023.06.1. Ranges of p values are indicated with asterisks: *p < 0.05; **p < 0.01; ***p < 0.001; ****p < 0.0001.

### Reporting summary

Further information on research design is available in the Nature Research Reporting Summary linked to this article.

### Supplementary information

Supplemental Figure 1

Reporting Summary

## Data Availability

The raw data supporting the conclusions of the study are available from the corresponding author.

## References

[1] Merad M, Blish CA, Sallusto F, Iwasaki A (2022). The immunology and immunopathology of COVID-19. Science.

[2] Osuchowski MF (2021). The COVID-19 puzzle: deciphering pathophysiology and phenotypes of a new disease entity. Lancet Respir. Med..

[3] Lamers MM, Haagmans BL (2022). SARS-CoV-2 pathogenesis. Nat. Rev. Microbiol..

[4] Chakraborty S, Mallajosyula V, Tato CM, Tan GS, Wang TT (2021). SARS-CoV-2 vaccines in advanced clinical trials: Where do we stand?. Adv. Drug Deliv. Rev..

[5] Voysey M (2021). Single-dose administration and the influence of the timing of the booster dose on immunogenicity and efficacy of ChAdOx1 nCoV-19 (AZD1222) vaccine: a pooled analysis of four randomised trials. Lancet.

[6] Sadoff J (2021). Safety and Efficacy of Single-Dose Ad26.COV2.S Vaccine against Covid-19. N. Engl. J. Med..

[7] Polack FP (2020). Safety and Efficacy of the BNT162b2 mRNA Covid-19 Vaccine. N. Engl. J. Med..

[8] Baden LR (2021). Efficacy and Safety of the mRNA-1273 SARS-CoV-2 Vaccine. N. Engl. J. Med..

[9] Krammer F (2020). SARS-CoV-2 vaccines in development. Nature.

[10] Vogel AB (2021). BNT162b vaccines protect rhesus macaques from SARS-CoV-2. Nature.

[11] Corbett KS (2020). SARS-CoV-2 mRNA vaccine design enabled by prototype pathogen preparedness. Nature.

[12] Mathieu, E. et al. *OurWorldInData.org* (2020).

[13] Sahin U (2020). COVID-19 vaccine BNT162b1 elicits human antibody and T(H)1 T cell responses. Nature.

[14] Widge AT (2021). Durability of Responses after SARS-CoV-2 mRNA-1273 Vaccination. N. Engl. J. Med..

[15] Tarke A (2022). SARS-CoV-2 vaccination induces immunological T cell memory able to cross-recognize variants from Alpha to Omicron. Cell.

[16] Garcia-Beltran WF (2022). mRNA-based COVID-19 vaccine boosters induce neutralizing immunity against SARS-CoV-2 Omicron variant. Cell.

[17] Nunez NG (2023). High-dimensional analysis of 16 SARS-CoV-2 vaccine combinations reveals lymphocyte signatures correlating with immunogenicity. Nat. Immunol..

[18] Andrews N (2022). Effectiveness of COVID-19 booster vaccines against COVID-19-related symptoms, hospitalization and death in England. Nat. Med.

[19] Pajon R (2022). SARS-CoV-2 Omicron Variant Neutralization after mRNA-1273 Booster Vaccination. N. Engl. J. Med..

[20] Dagan N (2021). BNT162b2 mRNA Covid-19 Vaccine in a Nationwide Mass Vaccination Setting. N. Engl. J. Med..

[21] Muik A (2021). Neutralization of SARS-CoV-2 lineage B.1.1.7 pseudovirus by BNT162b2 vaccine-elicited human sera. Science.

[22] Paschold L (2022). Rapid Hypermutation B Cell Trajectory Recruits Previously Primed B Cells Upon Third SARS-Cov-2 mRNA Vaccination. Front. Immunol..

[23] Reynolds CJ (2021). Prior SARS-CoV-2 infection rescues B and T cell responses to variants after first vaccine dose. Science.

[24] Lyke KE (2023). Immunogenicity of NVX-CoV2373 heterologous boost against SARS-CoV-2 variants. NPJ Vaccines.

[25] Springer DN (2023). Bivalent COVID-19 mRNA booster vaccination (BA.1 or BA.4/BA.5) increases neutralization of matched Omicron variants. NPJ Vaccines.

[26] Sitaras I (2022). Systematic review of primary and booster COVID-19 sera neutralizing ability against SARS-CoV-2 omicron variant. NPJ Vaccines.

[27] Brinkkemper M (2021). A third SARS-CoV-2 spike vaccination improves neutralization of variants-of-concern. NPJ Vaccines.

[28] Hall V (2022). Protection against SARS-CoV-2 after Covid-19 Vaccination and Previous Infection. N. Engl. J. Med..

[29] Barnes E (2023). SARS-CoV-2-specific immune responses and clinical outcomes after COVID-19 vaccination in patients with immune-suppressive disease. Nat. Med..

[30] Ehmsen S (2021). Antibody and T cell immune responses following mRNA COVID-19 vaccination in patients with cancer. Cancer Cell.

[31] Fendler A (2022). COVID-19 vaccines in patients with cancer: immunogenicity, efficacy and safety. Nat. Rev. Clin. Oncol..

[32] Mellinghoff SC (2022). SARS-CoV-2 specific cellular response following COVID-19 vaccination in patients with chronic lymphocytic leukemia. Leukemia.

[33] Keppler-Hafkemeyer A (2023). Potent high-avidity neutralizing antibodies and T cell responses after COVID-19 vaccination in individuals with B cell lymphoma and multiple myeloma. Nat. Cancer.

[34] Male V (2022). SARS-CoV-2 infection and COVID-19 vaccination in pregnancy. Nat. Rev. Immunol..

[35] Nalbandian A (2021). Post-acute COVID-19 syndrome. Nat. Med.

[36] Mehandru S, Merad M (2022). Pathological sequelae of long-haul COVID. Nat. Immunol..

[37] Altmann, D. M., Whettlock, E. M., Liu, S., Arachchillage, D. J. & Boyton, R. J. The immunology of long COVID. *Nat. Rev. Immunol.*10.1038/s41577-023-00904-7 (2023). 10.1038/s41577-023-00904-7 37433988

[38] Davis HE, McCorkell L, Vogel JM, Topol EJ (2023). Long COVID: major findings, mechanisms and recommendations. Nat. Rev. Microbiol..

[39] Nalbandian A, Desai AD, Wan EY (2023). Post-COVID-19 condition. Annu. Rev. Med..

[40] Brannock MD (2023). Long COVID risk and pre-COVID vaccination in an EHR-based cohort study from the RECOVER program. Nat. Commun..

[41] Al-Aly Z, Bowe B, Xie Y (2022). Long COVID after breakthrough SARS-CoV-2 infection. Nat. Med..

[42] Ayoubkhani D (2022). Risk of long COVID in people infected with severe acute respiratory syndrome coronavirus 2 after 2 doses of a coronavirus disease 2019 vaccine: community-based, matched cohort study. Open Forum Infect. Dis..

[43] Antonelli M (2022). Risk factors and disease profile of post-vaccination SARS-CoV-2 infection in UK users of the COVID Symptom Study app: a prospective, community-based, nested, case-control study. Lancet Infect. Dis..

[44] Azzolini E (2022). Association Between BNT162b2 Vaccination and Long COVID After Infections Not Requiring Hospitalization in Health Care Workers. JAMA.

[45] Diexer, S. et al. Association between virus variants, vaccination, previous infections, and post COVID-19 Risk. *Int. J. Infect. Dis.*10.1016/j.ijid.2023.08.019 (2023). 10.1016/j.ijid.2023.08.019 37634619

[46] Byambasuren O, Stehlik P, Clark J, Alcorn K, Glasziou P (2023). Effect of covid-19 vaccination on long covid: systematic review. BMJ Med..

[47] Schultheiss C (2022). The IL-1beta, IL-6, and TNF cytokine triad is associated with post-acute sequelae of COVID-19. Cell Rep. Med.

[48] Wisnivesky JP (2022). Association of vaccination with the persistence of post-COVID symptoms. J. Gen. Intern. Med..

[49] Wynberg E (2022). The effect of SARS-CoV-2 vaccination on post-acute sequelae of COVID-19 (PASC): A prospective cohort study. Vaccine.

[50] Tsuchida T (2022). Relationship between changes in symptoms and antibody titers after a single vaccination in patients with Long COVID. J. Med. Virol..

[51] Klein, J. et al. Distinguishing features of Long COVID identified through immune profiling. *medRxiv*, 2022.2008.2009.22278592 10.1101/2022.08.09.22278592 (2022). 10.1038/s41586-023-06651-y PMC10620090 37748514

[52] Evans RA (2021). Physical, cognitive, and mental health impacts of COVID-19 after hospitalisation (PHOSP-COVID): a UK multicentre, prospective cohort study. Lancet Respir. Med..

[53] Talla A (2023). Persistent serum protein signatures define an inflammatory subcategory of long COVID. Nat. Commun..

[54] Reese JT (2023). Generalisable long COVID subtypes: findings from the NIH N3C and RECOVER programmes. EBioMedicine.

[55] Schultheiss C (2023). Liquid biomarkers of macrophage dysregulation and circulating spike protein illustrate the biological heterogeneity in patients with post-acute sequelae of COVID-19. J. Med. Virol..

[56] Kervevan, J. et al. Divergent adaptive immune responses define two types of long COVID. *Front. Immunol.* 14 10.3389/fimmu.2023.1221961 (2023). 10.3389/fimmu.2023.1221961 PMC10408302 37559726

[57] Zhang H (2023). Data-driven identification of post-acute SARS-CoV-2 infection subphenotypes. Nat. Med..

[58] Etter MM (2022). Severe Neuro-COVID is associated with peripheral immune signatures, autoimmunity and neurodegeneration: a prospective cross-sectional study. Nat. Commun..

[59] Mateu, L. et al. Determinants of the onset and prognosis of the post-COVID-19 condition: a 2-year prospective observational cohort study. *The Lancet Regional Health – Europe*10.1016/j.lanepe.2023.100724 10.1016/j.lanepe.2023.100724 PMC10636281 37954002

[60] Lazarus JV (2023). A survey of COVID-19 vaccine acceptance across 23 countries in 2022. Nat. Med..

[61] Pierri F (2022). Online misinformation is linked to early COVID-19 vaccination hesitancy and refusal. Sci. Rep..

[62] Schultheiss C (2020). Next-Generation Sequencing of T and B Cell Receptor Repertoires from COVID-19 Patients Showed Signatures Associated with Severity of Disease. Immunity.

[63] Schultheiß, C. et al. Maturation trajectories and transcriptional landscape of plasmablasts and autoreactive B cells in COVID-19. *iScience*10.1016/j.isci.2021.103325 (2021). 10.1016/j.isci.2021.103325 PMC8536484 34723157

[64] Woodruff MC (2023). Chronic inflammation, neutrophil activity, and autoreactivity splits long COVID. Nat. Commun..

[65] Phetsouphanh C (2022). Immunological dysfunction persists for 8 months following initial mild-to-moderate SARS-CoV-2 infection. Nat. Immunol..

[66] Kovarik JJ (2023). A multi-omics based anti-inflammatory immune signature characterizes long COVID-19 syndrome. iScience.

[67] Altmann DM, Boyton RJ (2022). COVID-19 vaccination: The road ahead. Science.

[68] Sahin U (2021). BNT162b2 vaccine induces neutralizing antibodies and poly-specific T cells in humans. Nature.

[69] Jalkanen P (2021). COVID-19 mRNA vaccine induced antibody responses against three SARS-CoV-2 variants. Nat. Commun..

[70] Roltgen K (2022). Immune imprinting, breadth of variant recognition, and germinal center response in human SARS-CoV-2 infection and vaccination. Cell.

[71] Muik A (2022). Omicron BA.2 breakthrough infection enhances cross-neutralization of BA.2.12.1 and BA.4/BA.5. Sci. Immunol..

[72] Regev-Yochay G (2023). Correlates of protection against COVID-19 infection and intensity of symptomatic disease in vaccinated individuals exposed to SARS-CoV-2 in households in Israel (ICoFS): a prospective cohort study. Lancet Microbe.

[73] Gilbert PB (2022). Immune correlates analysis of the mRNA-1273 COVID-19 vaccine efficacy clinical trial. Science.

[74] Naranbhai V (2022). Neutralization breadth of SARS-CoV-2 viral variants following primary series and booster SARS-CoV-2 vaccines in patients with cancer. Cancer Cell.

[75] Kaneko N (2020). Loss of Bcl-6-Expressing T follicular helper cells and germinal centers in COVID-19. Cell.

[76] Turner JS (2021). SARS-CoV-2 mRNA vaccines induce persistent human germinal centre responses. Nature.

[77] Andrews N (2022). Covid-19 Vaccine Effectiveness against the Omicron (B.1.1.529) Variant. N. Engl. J. Med..

[78] Zollner A (2022). Postacute COVID-19 is Characterized by Gut Viral Antigen Persistence in Inflammatory Bowel Diseases. Gastroenterology.

[79] Sun J (2020). Prolonged persistence of SARS-CoV-2 RNA in body fluids. Emerg Infect Dis.

[80] Swank, Z. et al. Persistent circulating SARS-CoV-2 spike is associated with post-acute COVID-19 sequelae. *Clin. Infect. Dis.*10.1093/cid/ciac722 (2022). 10.1093/cid/ciac722 PMC10169416 36052466

[81] Noval Rivas M, Porritt RA, Cheng MH, Bahar I, Arditi M (2022). Multisystem Inflammatory Syndrome in Children and Long COVID: The SARS-CoV-2 Viral Superantigen Hypothesis. Front. Immunol..

[82] Porritt, R. A. et al. HLA class I-associated expansion of TRBV11-2 T cells in multisystem inflammatory syndrome in children. *J. Clin. Invest.* **131**10.1172/JCI146614 (2021). 10.1172/JCI146614 PMC8121516 33705359

[83] Vibholm LK (2021). SARS-CoV-2 persistence is associated with antigen-specific CD8 T-cell responses. EBioMedicine.

[84] Su Y (2022). Multiple early factors anticipate post-acute COVID-19 sequelae. Cell.

[85] Peluso, M. J. et al. Multimodal Molecular Imaging Reveals Tissue-Based T Cell Activation and Viral RNA Persistence for Up to 2 Years Following COVID-19. *medRxiv*, 2023.2007.2027.23293177 10.1101/2023.07.27.23293177 (2023).

[86] Proal, A. D. et al. SARS-CoV-2 reservoir in post-acute sequelae of COVID-19 (PASC). *Nat. Immunol*. 10.1038/s41590-023-01601-2 (2023). 10.1038/s41590-023-01601-2 37667052

[87] Gaebler C (2021). Evolution of antibody immunity to SARS-CoV-2. Nature.

[88] Natarajan A (2022). Gastrointestinal symptoms and fecal shedding of SARS-CoV-2 RNA suggest prolonged gastrointestinal infection. Med.

[89] Hu F (2020). A compromised specific humoral immune response against the SARS-CoV-2 receptor-binding domain is related to viral persistence and periodic shedding in the gastrointestinal tract. Cell Mol. Immunol..

[90] Garcia-Abellan J (2021). Antibody response to SARS-CoV-2 is associated with long-term clinical outcome in patients with COVID-19: a Longitudinal Study. J. Clin. Immunol..

[91] Lucke, J. et al. Intestinal IL-1beta Plays a Role in Protecting against SARS-CoV-2 Infection. *J. Immunol.*10.4049/jimmunol.2200844 (2023). 10.4049/jimmunol.2200844 PMC10476162 37556130

[92] Li Y (2021). Inflammasomes as therapeutic targets in human diseases. Signal Transduct. Target Ther..

