# Supplementary material for: SARS-CoV-2 vaccination may mitigate dysregulation of IL-1/IL-18 and gastrointestinal symptoms of the post-COVID-19 condition
Source: NPJ Vaccines. 2024 Feb 5;9:23. doi: 10.1038/s41541-024-00815-1 (PMC10844289; doi:10.1038/s41541-024-00815-1)
Supplement: Supplementary file 1 — Supplemental Figure 1 [file 41541_2024_815_MOESM1_ESM.pdf]

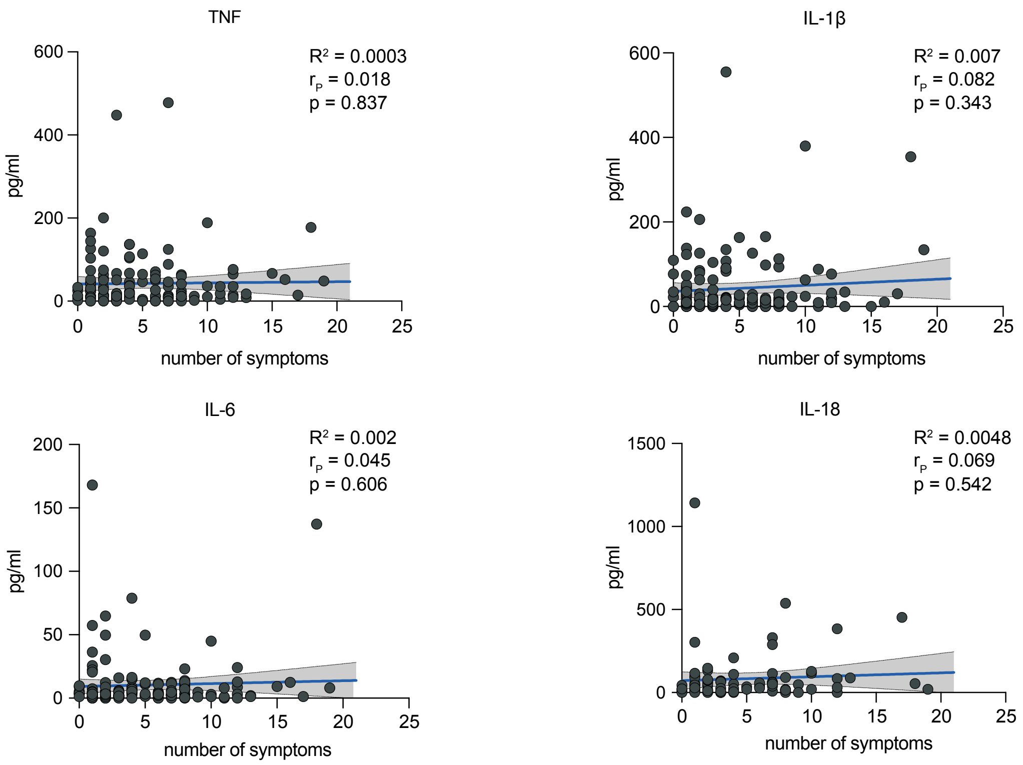

Supplementary Figure 1. Cytokine levels relative to number of reported symptoms in patients with ongoing PCC. Linear regression and Pearson correlation analysis for indicated plasma cytokine levels and number of symptoms reported in the online questionnaire of patients with ongoing PCC. Correlation coefficient R^2, Pearson correlation coefficients (r_P) and p values are indicated.
